# Hydrogen Bonding: Between Strengthening the Crystal Packing and Improving Solubility of Three Haloperidol Derivatives

**DOI:** 10.3390/molecules21060719

**Published:** 2016-06-01

**Authors:** Hardeep Saluja, Ahmed Mehanna, Riccardo Panicucci, Eman Atef

**Affiliations:** 1 Department of Pharmaceutical Sciences, Southwestern Oklahoma State University, 100 Campus Drive, Weatherford, OK 73096-3098, USA; hardeep.saluja@swosu.edu; 2 Department of Pharmaceutical Sciences, MCPHS-University-Boston, 179 Longwood Ave, Boston, MA 02115, USA; Ahmed.Mehanna@mcphs.edu; 3 WuXi AppTec, Cambridge, MA 02142, USA; rick.panicucci@wuxiapptec.com; 4 College of Pharmacy, California Northstate University, 9700 W Taron Drive, Elk Grove, CA 95757, USA

**Keywords:** drug crystal packing, hydrogen bonding, solid dispersion, haloperidol-related compounds, molecular interaction, hydrophobic interaction, droperidol, deshydroxyhaloperidol

## Abstract

The purpose of this study is to confirm the impact of polar functional groups on inter and intra-molecular hydrogen bonding in haloperidol (HP) and droperidol (DP) and, hence, their effects on dissolution using a new approach. To confirm our theory, a new molecule: deshydroxy-haloperidol (DHP) was designed and its synthesis was requested from a contract laboratory. The molecule was then studied and compared to DP and HP. Unlike DHP, both the HP and DP molecules have hydrogen donor groups, therefore, DHP was used to confirm the relative effects of the hydrogen donor group on solubility and crystal packing. The solid dispersions of the three structurally related molecules: HP, DP, and DHP were prepared using PVPK30, and characterized using XRPD and IR. A comparative dissolution study was carried out in aqueous medium. The absence of a hydrogen bonding donor group in DHP resulted in an unexpected increase in its aqueous solubility and dissolution rate from solid dispersion, which is attributed to weaker crystal pack. The increased dissolution rate of HP and DP from solid dispersions is attributed to drug-polymer hydrogen bonding that interferes with the drug-drug intermolecular hydrogen bonding and provides thermodynamic stability of the dispersed drug molecules. The drug-drug intermolecular hydrogen bond is the driving force for precipitation and crystal packing.

## 1. Introduction

Solubility and permeability are the most vital properties that determine the bioavailability of drugs upon oral administration, thus they form the basis of the biopharmaceutical classification system [1]. With the advent of newer technologies and the use of robotics in drug discovery, massive libraries of molecules are synthesized every year, however, many fail during early stages of pre-formulation due to poor biopharmaceutical properties [2]. Formulation scientists employ various approaches to address this issue. In addition to the classical approaches like particle size reduction, salt formation, co-solvency, and complexation, modern technologies are also used, such as: nanotechnology, micro emulsions and solid dispersions [3].

Amorphous drug forms may have multiple-fold higher solubility compared to their crystalline counterparts [3,4]. This approach is challenging, however, because the amorphous drug form is thermodynamically unstable, which results in frequent recrystallization upon storage. In solid dispersions, hydrophilic polymers have been utilized to stabilize the high energy amorphous form of drugs. These polymers offer a solubility advantage by improving wettability, and interfering with crystallization, thus maintaining the molecular dispersion of the drug in solution [5]. As a result, during the last decade, solid dispersions have drawn the attention of formulators as a new tool for improving the bioavailability of poorly-soluble drugs where solubility is limited by high crystallinity [6]. Solid dispersions, as with many non-traditional, relatively new techniques, have gaps in the knowledge base regarding its formulation [6]. This leads to poor predictability of factors that dictate dissolution rate and determine the stability of the drug in solid dispersion formulations. For example, there is a lack of profound understanding of the drug-polymer molecular interactions that govern the properties of solid dispersions, facilitate their optimization, and affect the stability of the incorporated drug and thus its *in vivo* performance [7,8].

Currently, in preparing solid dispersions, an empirical approach is used in polymer selection and the underpinning mechanisms involved in high dissolution using a specific polymer are not always discussed or rationalized [9]. Thus, there is a need to develop a rational assessment to determine whether the solubility and miscibility of certain drugs improves in the presence of polymers. This will allow for a more systematic, efficient process of polymer selection.

In addition, solid dispersions with fewer polymers demonstrate improved dissolution rates, but could not inhibit crystallization upon storage [10]. On the other hand, despite the ability to inhibit amorphous drug recrystallization upon storage, unchanged dissolution rates were observed in the cases of griseofulvin-PEG [11] and chlorothalidone-urea [12].

**Figure 1:**
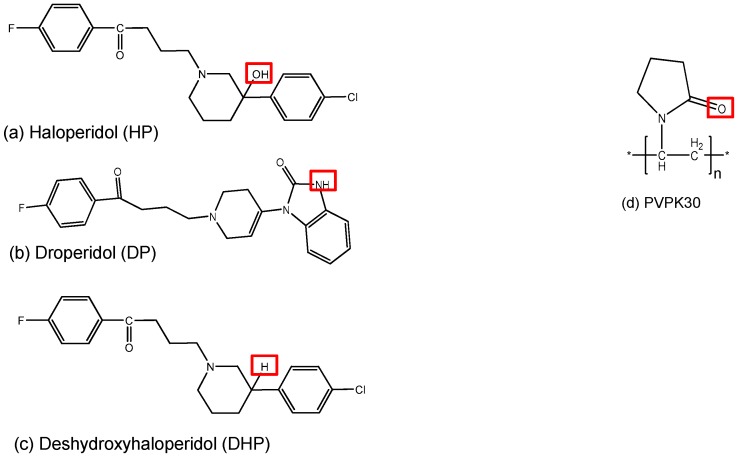
Structure of three model drugs (**a**–**c**) and the polymer (**d**) used in the study marking the hydrogen bonding donor and acceptor groups.

In order to address the preceding issues, it is important to thoroughly understand the mode of drug incorporation and its molecular interactions with the polymer. A profound understanding of the molecular behavior will provide an insight into the factors governing drug dissolution rates in the presence of a specific polymer. This could further aid in predicting the dissolution behavior of drugs with different polymers and the stability of the solid dispersion. The purpose of this study was to investigate the relative effect of hydrogen donor functional groups on drug-drug and drug-polymer intermolecular hydrogen bonding using three molecules with relatively similar structure and different or no hydrogen donor groups. In other words, to study the intermolecular hydrogen bonding effect on crystal packing *versus* solid dispersion stability. In this work we studied three structurally related molecules: haloperidol (HP), droperidol (DP) and deshydroxyhaloperidol (DHP), and their interactions with a hydrophilic polymer polyvinylpyrollidone K30 (PVPK30) (Figure 1). DHP was originally synthesized by Novartis (Basel, Switzerland).

Unlike DHP, both HP and DP have hydrogen donor functional groups—a hydroxyl functional group (-OH) and an amino group (-NH)—respectively. Both of these hydrogen donor groups can form hydrogen bonds with the carbonyl group (-C=O) on PVPK30. In the case of DHP, the hydroxyl and amino groups are absent, thus it is unable to form hydrogen bonds with PVPK30 or to form intermolecular hydrogen bonds, which could essentially affect crystal packing.

Solid dispersions were prepared using the solvent evaporation method and characterized using X-ray powder diffraction (XRPD) and differential scanning calorimetry (DSC). Fourier Transform Infrared Spectroscopy (FTIR) was performed to probe molecular interactions.

## 2. Results

### 2.1. Determination of Solubility Parameters

The average solubility parameters (δ) of PVPK30, HP, DP and DHP obtained from three different methods (Hansen’s 3D, Van Krevelen and Hoy’s Methods) were calculated and the difference between the solubility parameters of each drug and the polymer were determined. For example, the solubility parameter differences (∆δ) between PVPK30 and HP, PVPK30 and DP and PVPK30 and DHP were found to be 1.34, 1.80 and 0.48, respectively. If ∆δ is less than 4MPa1/2, it is likely that the two components will be miscible in each other [13]. Based on the miscibility results, summarized in Table 1, it was found that the three drugs were miscible with PVPK30. This was further confirmed using DSC.

### 2.2. DSC Characterization of the Drug Molecules

The DSC results are summarized in Table 2. A sharp melting endotherm was observed in HP at 151.2 °C in cycle 1 (Heat), recrystallization exotherm at 91.5 °C in cycle 2 (Cool) and a melting endotherm at 150.8 °C in cycle 3. The melting point observed in both cycle 1 and 3 were same, confirming that the drug recrystallized in its original crystal form after cooling the melt. On the other hand DP showed two melting endotherms at 145.2 and 152.1 °C in cycle 1 suggesting the existence of two crystal forms or the melting of a newly-formed crystal form transformed from the original counterpart. The first possibility is more likely though. No recrystallization exotherm was observed during cycle 2 (Cool) for DP. In cycle 3, a Tg was observed at 34.5 followed by a recrystallization exotherm at 103.6 °C and a melting endotherm at 149 °C, suggesting that the drug recrystallized as one crystal form [14].

A broad melting endotherm was observed in the DHP thermogram at 90.4 °C in cycle 1, a recrystallization peak at 21.2 °C in cycle 2, and two broad melting endotherms at 78.9 °C and 90.3 °C in cycle 3. This suggests that the DHP was originally present as a single crystal form, which recrystallized into two crystal forms. The above phenomenon can be explained as follows: the tight packing of HP, indicated by the high heat of fusion (148 J/g) is due to hydrogen bonding between the hydroxyl and the carboxyl functional groups. The amino functional group in DP represents a weaker hydrogen bonding donor group than the hydroxyl group, resulting in looser crystal packing than HP. This is confirmed by the lower heat of fusion (109 J/g) of DP.

When subjected to heat cool heat cycles in DSC, DP did not recrystallize on cooling (cycle 2) and showed a Tg in the third cycle, suggesting the existence of a relatively stable amorphous form of the drug during cooling. DHP lacks the hydrogen donor functional groups necessary to form intermolecular hydrogen bonds in a solid state reflected by the melting temperature and enthalpy of fusion of DHP, which were almost half that of HP.

The Tg of HP and DHP could not be determined experimentally due to the rapid recrystallization during the cooling cycle. The amorphous state of HP and DHP could not be generated using either quench cooling, solvent evaporation or lyophilization, thus Tg for HP and DHP was calculated based on the empirical relationship Tg = 0.7 Tm K (Kelvin). The calculated Tg is an estimated rough value [5]. A single Tg was observed with all binary mixtures with PVPK30 at different percentage drug load (DL), suggesting drug polymer miscibility.

### 2.3. Solid Dispersions Preparation

#### 2.3.1. XRPD Characterization of the Solid Dispersions

**Figure 2:**
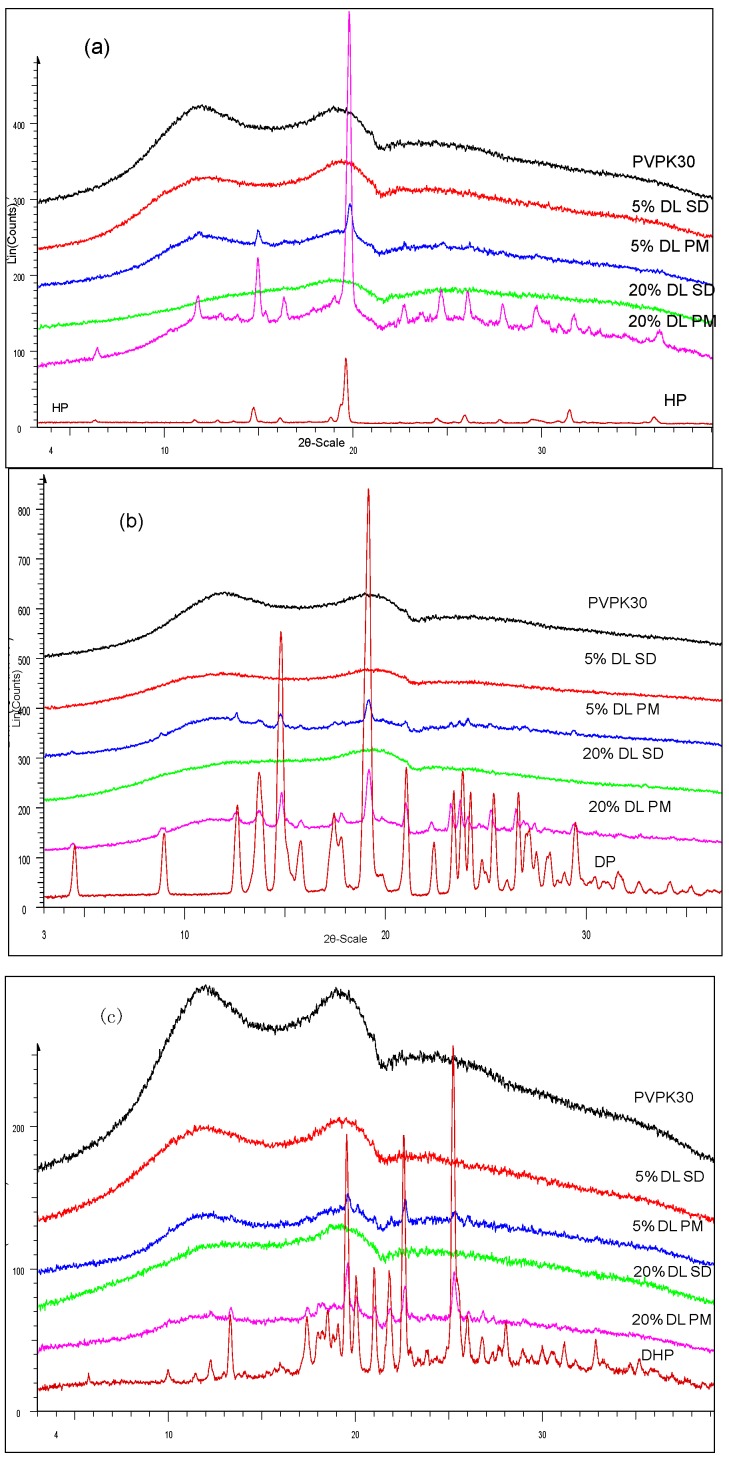
XRPD of (**a**) HP; (**b**) DP; (**c**) DHP with PVPK30 solid dispersion (SD) and physical mixture (PM). The drugs found amorphous in solid dispersion and crystalline in physical mixture.

Results confirmed the amorphous state of HP, DP and DHP in solid dispersions in the presence of PVPK30 (Figure 2a–c).

#### 2.3.2. IR Characterization of the Solid Dispersions

Table 3 summarizes the peak assignments of studied HP, DHP and DP functional groups. The FTIR spectra of HP and DHP show a carbonyl group characteristic peak at 1680 cm^−1^. The HP also shows a characteristic hydroxyl group peak at 3100 cm^−1^ assigned to hydrogen bound hydroxyl groups [15]. The complete amorphous form of HP and DHP could not be generated and the IR spectra for partly crystalline and crystalline forms of HP and DHP were found to be similar. 

**Figure 3:**
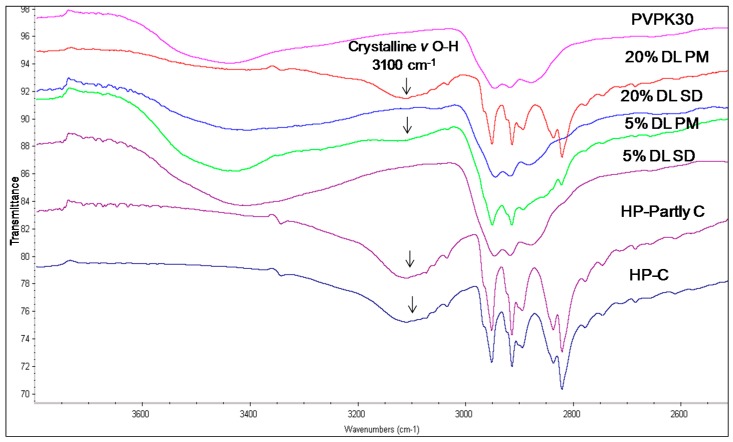
FTIR spectra of haloperidol PVPK30 solid dispersion (SD) and physical mixture (PM) showing O-H stretching region at 3100 cm^−1^ in crystalline (C), partly crystalline (partly C) and physical mixture (PM), which disappears in solid dispersion (SD) at both 5% and 20% drug loading (DL).

**Figure 4:**
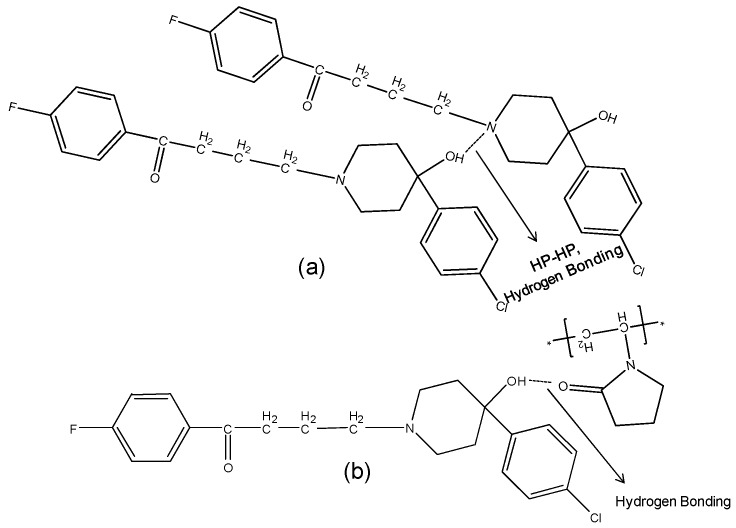
Schematic representation of (**a**): HP-HP Intermolecular hydrogen bonding in haloperidol. The two molecules are bonded through hydrogen bonding between -OH of molecule with -N of other; (**b**): Hydrogen bonding and dipole-dipole interaction between HP and PVPK30 solid dispersion.

The hydroxyl group peak at 3100 cm^−1^ disappears in solid dispersions, whereas it remains in physical mixtures (Figure 3); confirming a change in the environment of the hydroxyl group, potentially due to drug-polymer hydrogen bonding interaction. According to the HP single crystal data [16] the unit cell consists of two hydrogen bonded molecules. The bond is formed between the –OH of one molecule and the -N of the other leading to tight crystal packing. A schematic representation is shown in Figure 4a).

When the HP-PVPK30 solid dispersion is formed, the crystalline HP gets converted to an amorphous form as confirmed by XRPD (Figure 2a). The HP-HP intermolecular hydrogen bonds are broken and replaced by a hydrogen bond with the polymer’s carbonyl group, resulting in the disappearance of the hydroxyl peak at 3100 cm^−1^ in solid dispersion (Figure 3 and Figure 4a,b).

**Figure 5:**
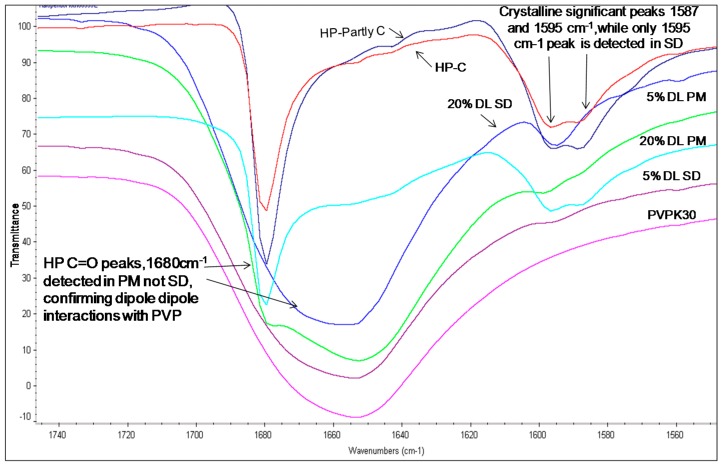
FTIR spectra of HP-PVPK30 solid dispersion (SD) and physical mixture (PM) (1680 cm^−1^) showing -C=O stretching region at 1680 cm^−1^ in crystalline, (C) partly crystalline and physical mixture, which shifts in SD.

**Figure 6:**
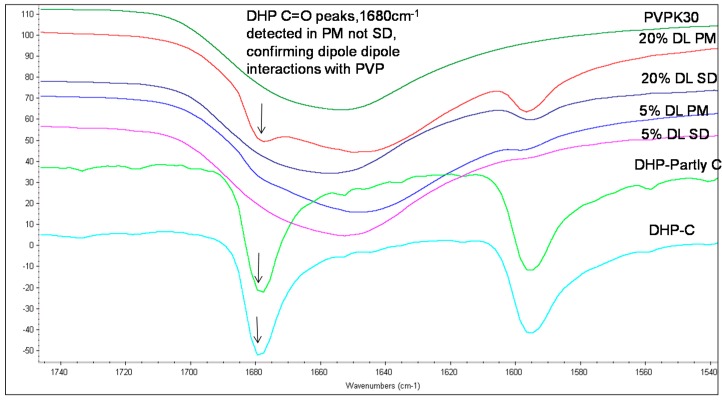
FTIR spectra of DHP PVPK30 solid dispersion (SD) and physical mixture (PM) showing ‑C=O stretching region at 1680 cm^−1^ in crystalline (C), partly crystalline and physical mixture, which shifts in solid dispersion.

The carbonyl group peaks of HP and DHP at 1680 cm^−1^ could be detected in the physical mixtures but not in the solid dispersions (Figure 5 and Figure 6).

The fingerprint region above 1600 cm^−1^ in the HP solid dispersion was found to be different than the physical mixture. For example, two peaks were seen in the crystalline drug and physical mixture at 1595 cm^−1^ and 1587 cm^−1^, while in the solid dispersion, only a single peak at 1590 cm^−1^ was observed. This further suggests changes in the molecular environment of the drug in solid dispersion compared to the physical mixture, confirming drug polymer interaction in the solid state (Figure 5).

**Figure 7:**
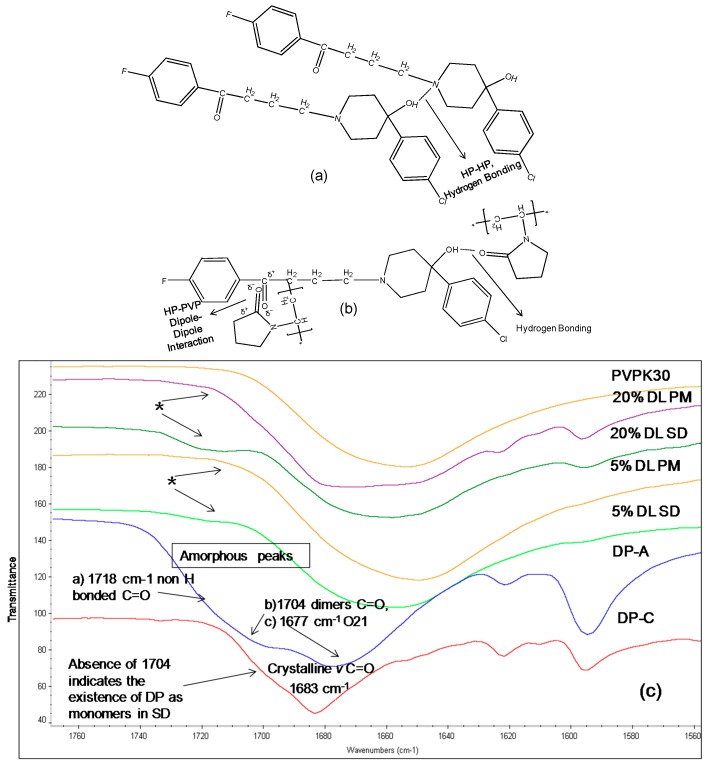
(Top **a**,**b**): Schematic representation of (**a**): Intermolecular hydrogen bonding in DP. Two molecules are linked together by two hydrogen bonds, linking the benzimidazole terminals through N8H and O1 atoms (**b**): Hydrogen bonding and dipole dipole interaction between DP and PVPK30 (bottom **c**) FTIR spectra of droperidol (DP), crystalline (C), amorphous(A), PVPK30 solid dispersion (SD) and physical mixture (PM) at 1680 cm^−1^ 1677 cm^−1^ (O21), 1704 cm^−1^ (C=O, (O1) in dimers) and 1718 cm^−1^ (non-hydrogen bonded C=O, (O1)). The 1704 cm^−1^ correspond to O1 in dimer structure. * The peak disappearing in the solid dispersions indicating the lack of the dimers in the solid dispersion.

The IR spectrum of crystalline DP shows a characteristic carbonyl group peak at 1683 cm^−1^. In the amorphous form, the carbonyl peak at 1683 cm^−1^ splits into two broad peaks and a shoulder (Figure 6c). DP exists as a dimer linked together by two hydrogen bonds between the benzimidazole terminals through the N8H and O1 atoms (Figure 7a,c) [15]. When converted to an amorphous form, the intermolecular hydrogen bonds break, resulting in two peaks and a shoulder in the IR spectra at 1677 cm^−1^ (O21), 1704 cm^−1^ (C=O, (O1) in dimers) and 1718 cm^−1^ (non-hydrogen bonded C=O, (O1)) [17].

The absence of the peak at 1704 cm^−1^ in solid dispersion suggests that the drug molecules predominantly exist as monomers. The carbonyl peak of the drug at O21 was found to disappear in solid dispersion both at 5% and 20% DL; however the crystalline form characteristic peak of the drug was clearly detected at 1683 cm^−1^ in 20% DL physical mixture. This suggests possible dipole-dipole interaction between -C=O of polymer with –C=O of drug (at O21) in solid dispersion as described in (Figure 7b).

### 2.4. Single Crystal X-ray

**Figure 8:**
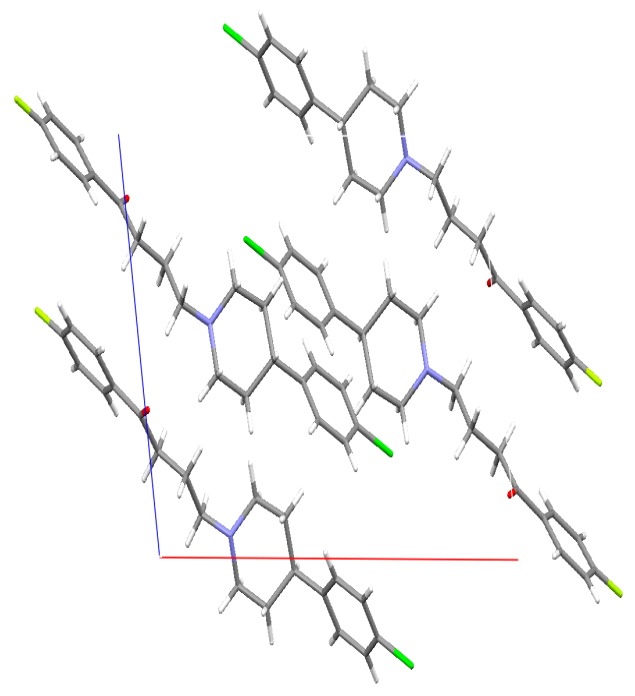
Crystal structure of deshydroxyhaloperidol in the unit cell. X-ray diffraction experiments were performed on single crystals with an Xcalibur 3 four-circle diffractometer (Oxford Diffraction). For details, see Supplementary Crystallographic Data.

The crystal structure of DHP was determined at Novartis. The data showed the presence of four molecules of DHP per unit cell. The four DHP molecules are held together by weak van der Waals forces, which was anticipated due to the lack of the hydroxyl functional group (Figure 8).

The crystal shown in the figure above is the deshydroxyhaloperidol (DHP). The CIF indicated that there is a possible 20% of the synthesized (DHP) mixture is the non-chlorinated analogue. Although this can result is changes in the dissolution as the chlorine atom can result in partial changes in the solubility of the DHP, but the authors thinks that the major observed changes in the DHP are due to the removal of the hydroxyl group from the HP, which was observed in 100% of the molecules.

In DSC when the mixture of 80% DHP and 20% non-chlorinated analogue are melted: in cycle one the mp is 90.4 °C and in cycle 3 there was two endotherms: one major peak at 90.4 °C and a shoulder at 77.5 °C, which may be due to the separation of the non-chlorinated analogue. The above suggests that the presence of non-chlorinated analogue didn’t affect the melting point of the major component DHP as it remained exactly the same in cycle one and three (90.4 °C).

### 2.5. Solubility Studies

**Figure 9:**
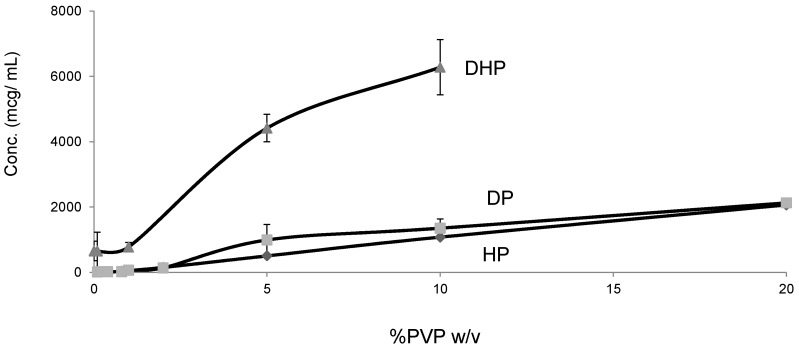
Equilibrium solubility of HP, DP and DHP at varying concentration of PVPK30 in water.

Unlike HP and DP, DHP exhibited high equilibrium solubility and dissolution in water (200 mcg/mL), which is approximately 20 times higher than the values for HP and DP (Figure 9). This unexpected increase in solubility, despite the decreased polarity, can be attributed to the weak intermolecular bonds, which results in weak crystal packing of DHP and is confirmed by the low enthalpy of crystallization (Table 2).

For a crystalline drug to dissolve in water, it is imperative to disrupt the crystal lattice with solvent molecules. In the case of DHP, the molecules in the crystal lattice are more loosely packed than HP, which is confirmed by the single crystal structure of DHP (Figure 8). In addition, the DSC results showed a decrease in the melting temperature and enthalpy of fusion of DHP compared to HP and DP (Table 2).

**Figure 10:**
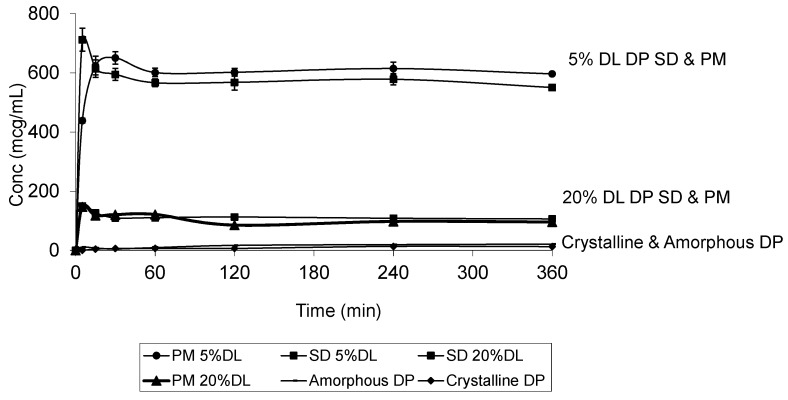
Dissolution profile of crystalline and amorphous DP, DP-PVPK30 solid dispersion (SD) and physical mixture (PM) at 5% and 20% drug loading (DL) confirming that the dissolution is dependent on the polymer concentration and not the drug form.

The DP dissolution profiles show clear polymer concentration dependent solubility enhancement. The initial drug crystal form has absolutely no effect on the solubility. Figure 10 shows that amorphous DP has no dissolution advantage over the crystalline form. This is confirmed by the similarity of the DP dissolution profiles from solid dispersions and physical mixtures at both 5% and 20% DL. The above also confirms that the polymer effect on DP solubility is predominantly due to its dissolution enhancement, not its amorphous state stabilization. On the other hand, the solubility of DHP and HP are dependent on both the polymer concentration and physical form. This is true at different DL.

The 100% amorphous forms of DHP and HP are unattainable, which reflects thermodynamic instability and an ease of recrystallization. Thus, the amorphous forms of both DHP and HP were unachievable and there were clear similarities between the patterns of the dissolution profiles of DHP and HP.

**Figure 11:**
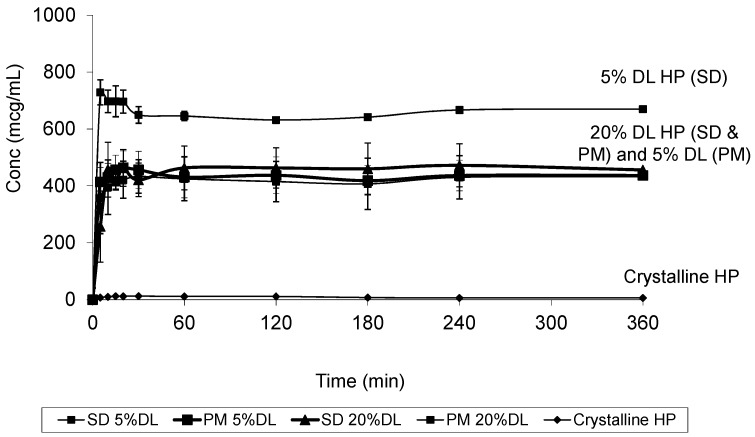
Dissolution profile of crystalline HP, HP PVPK30 solid dispersion (SD) and physical mixture (PM) at 5% and 20% drug loading (DL).

**Figure 12:**
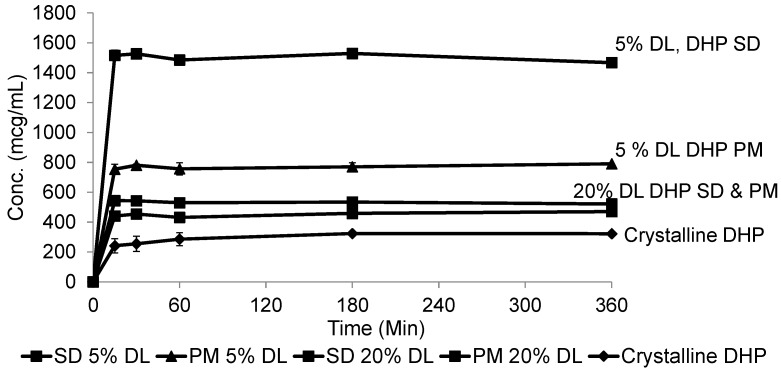
Dissolution profile of crystalline DHP, DHP-PVPK30 solid dispersion (SD) and physical mixture (PM) at 5% and 20% drug loading (DL).

At 20% DL the improved dissolution of the SD compared to the PM, was more dependent on polymer concentration than the solid crystal form. It is clear from (Figure 11 and Figure 12) that the release from PM and SD are very similar. The above trend is clear in both DHP and HP, despite the absence of polymer-DHP hydrogen bonding.

At low drug load, HP dissolution profiles were polymer concentration dependent, which is as an intermediate scenario between DHP and DP. The loss of the hydroxyl group resulted in less packed DHP crystals and weaker intermolecular bonds, confirmed by the low enthalpy of fusion and the crystal structure. The weak intermolecular bonds in DHP allow for higher kinetic solubility and this effect is further improved by the presence of PVP polymer, which prevents recystallization. The absence of hydrogen donor groups also affects the molecule charge distribution, and consequently the crystals packing. This explains the weak crystal structure of DHP. Although studying the drugs’ behavior from solid dispersions is important to formulating successful stable amorphous forms, the major benefit is not fully achieved until the stable solid state is translated into an improved solubility and bioavailability. HP and DP are interesting molecules to use in running comparative solid dispersion and dissolution studies, because the two molecules are structurally related and both have poor inherent solubility. HP exists in one crystal form and could not be prepared in amorphous form, yet DP has at least two crystal forms and can be successfully prepared as an amorphous form.

Considering the structure of both HP and DP, it’s reasonable to expect that HP-HP and DP-DP intermolecular hydrogen bonds do exist between similar molecules in the crystalline drug form. It has also been reported that the π-stacking interaction exists [18]. It is also reasonable to expect that a polymer with a carbonyl group like PVP K30 would be able to form hydrogen bonds with both molecules, thus stabilizing the drugs’ amorphous forms and improving their solubility. There were other polymers that were reported to be more efficient in stabilizing HP, but PVPK30 was the best choice for the purpose of our study as it has a hydrogen acceptor but not a donor functional group.

HP was found, as expected, to have closer packing due to the fact that the hydroxyl group of HP forms stronger intermolecular hydrogen bonds than the amino groups of DP. This was confirmed by the observed lower enthalpies of fusion and re-crystallization of DP compared to HP.

To further study the effect of hydrogen bond donor groups on the solubility and crystal packing of HP and DP, we added a newly synthesized DHP to our study. Unlike DP and HP, DHP lacks the hydrogen donor functional group. The expectation was that DHP, with less polarity, will have lower solubility than both HP and DP. But, surprisingly, we found out that the synthesized compound has significantly higher solubility and exists as a low melting point crystalline form. It is obvious that the crystal packing is dominated by weak van der Waals intermolecular interactions, thus we can also conclude, due to the structural similarity, that in addition to the hydrogen bonding in HP and DP crystals, weak van der Waals intermolecular interactions have a predominant role in crystal packing.

Among the three molecules, only DP was successfully prepared as an amorphous form. This was confirmed by another group of scientists [19]. The DP was classified as a class II crystallization compound, which means that the compound lacks crystallization during cooling from an undercooled melt state. These molecules have high glass forming ability and are crystalline in nature. In other words, they exist in a crystalline form but it is possible to prepare them in an amorphous form. On the other hand, HP and DHP are classified as class I molecules, that is hard to prepare as a pure amorphous form without excipients or polymers. Looking at the three molecules’ chemical structures, it is clear that the common fluorophenyl ring is not the driving force for this crystallization classification. Otherwise, the three molecules would have had identical crystallization behavior [20,21]. As the only differences between the three molecules are the chlorophenyl ring and the benzimidazole ring, it is reasonable to assume that the chlorophenyl ring, found in HP and DHP, facilitates crystallization, while the DP molecule exits in an amorphous form due to its distinctive benzimidazole ring.

The crystal packing of DP is dominated by van der Waals forces which explains the existence of DP in more than 10 solvates and the inability of DP to pack efficiently results in solvent voids [22]. This same argument could be used to explain the nonspontaneous crystallization of DP, which allows for successful preparation of the drug as an amorphous form.

For crystallization to occur, the molecules need to be in a particular orientation, and nucleation and, thus, crystallization occur faster when the probability of that requisite orientation is higher. The chlorophenyl moiety in HP and DHP, is a symmetric group around a rotatable bond, and, thus, exists in the right conformation or orientation for crystallization more often than the benzimidazole group in DP. The same concept explains how rigid molecules without free rotation bonds have higher crystallinity [19].

DHP is more soluble than HP and DP even though it is less polar, therefore we can conclude that the -OH and -NH polar groups have a less prominent effect on the intrinsic solubility of DP and HP than on the intermolecular hydrogen bonding of HP-HP and DP-DP. Thus, removal of the hydrogen bonding donor polar groups (in DHP) resulted in decreased crystal packing and increased solubility. This is confirmed through the observed dropping of the melting points and the enthalpy of fusion.

Thus, both the existence of the hydrogen bonding groups and the probability of the molecules to exist in a crystallization favorable orientation govern the crystal packing and the likelihood of creating an amorphous form of the drug. The same drug (D-D) hydrogen bonding can also impact the success of solid dispersions formulations. In other words if the drug-polymer intermolecular bonds are weaker than the corresponding D-D, the polymer will not be able to interfere with crystallization [22,23], which was not the case with the studied three molecules.

## 3. Materials and Methods

### 3.1. General Information

The HP, DP, and PVPK30 were purchased from Sigma Aldrich Co., (St. Louis, MO, USA). The DHP was synthesized by Best West laboratories, (Salt Lake City, UT, USA). The dichloromethane and ethanol of analytical grade were obtained from Fisher Scientific (Pittsburgh, PA, USA).

### 3.2. Preparation of Solid Dispersions and Physical Mixtures

Solid dispersions at 5% and 20% drug loading (DL) were prepared using the solvent evaporation method. The drug and polymer were dissolved in a mixture of dichloromethane and ethanol at a ratio of 1:1. The solvent was evaporated using a rotary evaporator (Buchi rotavapor R 200 series, New Castle, DE, USA) immersed in a water bath maintained at 50 °C. The prepared samples were dried in a vacuum oven held at 37 °C for 48 h to remove residual solvent. The obtained glassy dispersion was scraped, lightly triturated, and sieved. These triturated solid dispersions were characterized by XRPD (Bruker AXS-XRD, Billerica, MA, USA) and DSC Q2000 (TA Instruments, New Castle, DE, USA) after preparation to ensure the complete amorphization of the crystalline drug and were used immediately in solubility studies. The physical mixtures (PM) of the drugs and the polymer were prepared using geometric dilution.

### 3.3. Solubility Studies

Polymer solutions of varying concentrations were prepared, and the equilibrium solubility of each drug was determined. An excess of drug was mixed with 5 mL of the polymer solutions pre-equilibrated at 37 ± 0.5 °C (*n* = 3). The vials were shaken mechanically in a water bath shaker for 24 h; samples were filtered through 0.45 µm nylon filters. The samples were diluted adequately, and drug concentration was analyzed using Agilent 1100 HPLC (Hewlett Packard, Palo Alto, CA, USA).

### 3.4. Single Crystal Structure Determination

X-ray diffraction experiments were performed on single crystals with an Xcalibur 3 four-circle diffractometer (Oxford Diffraction, UK). We used an Oxford Cryosystems nitrogen-flow cryostat for measurements down to 80 K, and an Oxford Diffraction Helijet helium-flow cryostat for measurements at 15 K. The unit-cell parameters and data reduction were obtained with the Mercury software 3.1 (The Cambridge Crystallographic Data Centre, Cambridge, UK) from Oxford Diffraction. The structures were solved by direct methods (SIR-97) and refined against F2 by full-matrix least-squares techniques (SHELXL-97) with anisotropic displacement parameters for non-hydrogen atoms.

### 3.5. X-ray Power Diffraction 

A small amount of sample was placed on a glass slide and scanned using XRPD on a D8 Advance instrument (Bruker, Madison, MI, USA) for solid state characterization. The scanning was done over a range of 3–50° 2θ for 120 s. The samples were analyzed using EVA software. The diffraction patterns of solid dispersions were compared to the drug, polymer and physical mixtures.

### 3.6. Dissolution Test

Dissolution testing of the solid dispersions and the physical mixtures was carried out in distilled water. During the dissolution testing experiments the drug weight was kept constant. In other words, the total weight of the tested SD and PM with different drug loads was variable but the amount of drug remained the same in all samples. The drug was mixed with 5 mL of distilled water (37 ± 0.5 °C). The mixture was stirred using a rotating magnetic stirrer at 200 rpm. Either the solid dispersion or physical mixture was added to the vial and was analyzed for solubilized drug content after 5, 15, 20, 30, 60, 120, 180, 240, and 360 min. The samples were analyzed using an Agilent 1100 HPLC Chromolith column and data were analyzed using Empower 2 software version 6.10 (NGMSI, Waters Eschborn, Germany).

### 3.7. Thermal Analysis

Thermal Analysis was carried out using a Differential Scanning Calorimeter, DSCQ2000 (TA Instruments). The thermal events were quantified using universal analysis 2000 version 3.9A software (TA Instruments). The compounds were subjected to heat-cool-heat cycles. In the first cycle (cycle 1), the compounds were heated at a constant rate of 5 °C/min above their melting points but below their degradation temperatures as determined by thermogravimetric analysis (TGA). In the second cycle (cycle 2) the compounds were cooled and reheated in the third cycle (cycle 3). The thermal events such as glass to rubber transition, recrystallization and melting were detected and quantified. All the solid dispersions and physical mixtures were analyzed using DSC. 

### 3.8. Solubility Parameter Calculations

The solubility parameters were used to correlate miscibility of the drug and the polymer systems:

δ = (CED) 0.5 = (∆Ev/Vm) ^0.5^ (1)

where δ is the Hildebrand solubility parameters, CED is the cohesive energy per unit volume, ∆Ev is the energy of vaporization and Vm the molar volume.

The cohesive energy of a material was calculated based on the group contribution method using Molecular Modeling pro software version 6.3.1 (NGMSI, North Wales, PA, USA). The solubility parameters were calculated using the Van Krevelen, Hansen’s 3D solubility and Hoy’s methods [24].

### 3.9. Fourier Transform Infrared Spectroscopy (FTIR)

FTIR was performed to investigate the drug-polymer intermolecular interactions. We focused mainly on 3000 cm^−1^ to 3400 cm^−1^ and 1600 cm^−1^ to 1700 cm^−1^ regions corresponding to hydroxyl and carbonyl groups’ vibrational regions respectively. The IR spectra were collected using Nicolet 2000 (Thermo Fisher Scientific, Walkersville, MD, USA). Thirty two scans were collected at a resolution of 4 cm^−1^ for each sample over a wave number region of 400 to 4000 cm^−1^. The OMNIC software version 7.2a (Thermo Electorn, Waltham, MA, USA) was used for spectral analysis.

## 4. Conclusions

Hydrogen bonding has two opposite and crucial roles: it helps in stabilizing the pure drug crystal structure, which hinders the drug dissolution and it also stabilizes the drug- polymer solid dispersion which improves the drug dissolution rate. The absence of a hydrogen donor group in a drug molecule and thus the lack of intermolecular hydrogen bonding and the lack or reduction in molecular charges can significantly weaken the drug crystal packing and augment its intrinsic solubility. Thus the net effect of the polar groups on the dissolution of molecules is governed by multiple factors and therefore the removal of polar group can increase or decrease the drug kinetic solubility and affect the dissolution advantage from solid dispersions.

## Figures and Tables

**Table 1:** The solubility parameters and solubility parameter differences (δ) of PVPK30 and the three studied molecules. The solubility parameters were used in predicting the polymer/drugs miscibility.

Solubility Parameter Comparison						
Polymer/Drugs	Hansen’s 3D Method	Van Krevelen Method	Hoy’s Method	Average	Difference (δ)	Classification
PVPK30	21.57	22.18	22.95	22.23		
Haloperidol	23.09	25.15	22.47	23.57	1.34	Miscible
Droperidol	23.63	25.09	23.39	24.04	1.80	Miscible
Deshydroxyhaloperidol	20.90	22.68	21.69	21.76	0.48	Miscible

**Table 2:** Summary of Thermal Events of HP, DP, DHP. Cycle 1 = Heat, Cycle 2 = Cool, Cycle 3 = Reheat. Recrys. = recrystallization, M.p. = melting point.

Drug	M.p., Tm °C (Cycle 1)	Enthalpy Fusion ∆H (J/g) (Cycle 1)	Tg (°C) (Cycle 1)	Recrys. Temperature Tc (°C) (Cycle 2)	Enthalpy Recrys ∆H (J/g) (Cycle 2)	Recrys Temperature Tc (°C)/Melting Point Tm (°C) (Cycle 3)	Enthalpy Recrys ∆H (J/g)/Enthalpy Fusion ∆H (J/g) (Cycle 3)	TGA (°C)	* Calculated Tg (°C)
HP	151.2 ± 1.7	148 ± 1.2	-	91.5 ± 1.7	100.3 ± 3.3	150.8 ± 0.9	136.4 ± 1.2	>240	23.8
DP	145.2 ± 1.1/ 152.1 ± 0.2	109 ± 6.8				34.5 ± 0.9 (Tg) 103.6 ± 4.9 (Tc) 149.1 ± 1.4 (Tm)	60.8 ± 1.7 (∆H, recrys) 64.8 ± 15.6 (∆H, melting)	>270	-
DHP	90.4 ± 2.8	77 ± 1.7	-	21.2 ± 5	51.1 ± 3.3	78.9 ± 1.2/ 90.3 ± 0.6	11 ± 7.6/ 15.1 ± 7.4	>200	*-18.55

* the calculated Tg of DHP came up to a negative value, much lower than HP, due to the absence of hydroxyl groups.

**Table 3:** Summary of infrared spectral peaks for HP, DP, DHP- PVPK30 binary systems studied.

Drug/Polymer	Functional Group	Crystalline (cm^−1^)	Amorphous	Solid Dispersion	Physical Mixture (cm^−1^)
Haloperidol	H-bonded hydroxyl (OH)	3100	-	Disappears	3100
	Carbonyl (C=O)	1680	-	Disappears	1680
Deshydroxyhaloperidol	Carbonyl (C=O)	1680	-	Disappears	1680
Droperidol	Carbonyl (C=O)	1683	1677 (O21)	Disappears	1683
			1704 (O1 in dimer)	* Disappears	-
			1718 (O1 non H-bonded)	1720	-

* The peak disappearing in the solid dispersions indicating the lack of the dimers in the solid dispersion.

## Supplementary Materials

The CIF file can be available upon request.

## Abbreviations

The following abbreviations are used in this manuscript:

∆Ev: Energy of vaporization
Vm: Molar Volume
δ: Difference
°C: Degree Celsius
%: Percent
HP: Haloperidol
DP: Droperidol
DHP: Deshydroxy-haloperidol
DL: Drug Loading
XRPD: X ray Powder Diffraction
DSC: Differential Scanning Calorimetry
FTIR: Fourier Transform Infrared Spectroscopy
